# Cilia-associated cellular function of the AAA ATPases RUVBL1 and RUVBL2

**DOI:** 10.1186/2194-7791-2-S1-A29

**Published:** 2015-07-01

**Authors:** Claudia Dafinger, Markus Rinschen, Carolin Ehrenberg, Mareike Franke, Heike Göbel, Jörg Dötsch, Thomas Benzing, Bernhard Schermer, Max Christoph Liebau

**Affiliations:** Division of Pediatric Nephrology and Center for Experimental Research in Pediatric Nephrology, Department of Pediatrics, University Hospital of Cologne, Germany; Nephrology Research Laboratory, Department of Internal Medicine II, University Hospital of Cologne, Germany; Department of Radiology, University Hospital of Cologne, Germany; Institute of Pathology, University Hospital of Cologne, Germany; Center for Molecular Medicine, University Hospital of Cologne, Germany

## Meeting abstract

Genetic cystic kidney diseases, including autosomal recessive polycystic kidney disease (ARPKD), are among the most common causes of end stage renal disease in children and in adolescents. While the detailed biological events resulting in cyst formation remain incompletely understood, nowadays there is wide agreement that dysfunction of a specialized cellular organelle, the primary cilium, underlies genetic cystic kidney disease. In addition to cystic kidney disease, phenotypes resulting from impaired ciliary function can affect nearly every organ of the human body and are nowadays termed ciliopathies.

We recently identified the AAA ATPase RUVBL1 as part of various ciliopathy-associated renal protein complexes and could show that loss of *Ruvbl1* in the renal tubule leads to a severe ARPKD-like cystic kidney phenotype in mice. RUVBL1 has previously been linked to regulation of cilia-associated signaling pathways.

To obtain novel insights into the cellular function of RUVBL1 and its partner protein RUVBL2, we generated stable murine cell lines with single genomic integration of coding sequences for fluorescence-tagged Ruvbl1 and Ruvbl2. Out of these cell lines we performed protein interaction screens by repetitive and independent immunoprecipitations followed by quantitative mass spectrometry. Along with multiple known interaction partners we identified novel candidates including proteins that had already been linked to ciliary function. In addition to the important role of RUVBL1 in the renal tubule our data point to a more general function of the RUVBL proteins and the RUVBLs-containing chaperone-like R2TP protein complex for ciliary function. Novel murine *in vivo* data strongly supports this concept.

In summary, we obtained novel functional insights into the cell biological link of the AAA ATPases RUVBL1 and RUVBL2 to primary cilia. Our data suggest a role of the RUVBL proteins in the cytosolic pre-assembly of ciliary protein complexes.

